# Controlling Access to Suicide Means

**DOI:** 10.3390/ijerph8124550

**Published:** 2011-12-07

**Authors:** Marco Sarchiapone, Laura Mandelli, Miriam Iosue, Costanza Andrisano, Alec Roy

**Affiliations:** 1 Department of Health Sciences, University of Molise, Via De Sanctis, Campobasso 86100, Italy; Email: marco.sarchiapone@me.com (M.S.); m.iosue@alice.it (M.I.); 2 Institute of Psychiatry, University of Bologna, 5 V.le C. Pepoli, Bologna 40133, Italy; Email: costanza.andrisano@studio.unibo.it; 3 Psychiatry Service, Department of Veterans Affairs, 385 Tremont Ave., East Orange, NJ 07018, USA; Email: alec.roy@va.gov

**Keywords:** suicide, means of suicide, restriction of means

## Abstract

*Background*: Restricting access to common means of suicide, such as firearms, toxic gas, pesticides and other, has been shown to be effective in reducing rates of death in suicide. In the present review we aimed to summarize the empirical and clinical literature on controlling the access to means of suicide. *Methods*: This review made use of both MEDLINE, ISI Web of Science and the Cochrane library databases, identifying all English articles with the keywords “suicide means”, “suicide method”, “suicide prediction” or “suicide prevention” and other relevant keywords. *Results*: A number of factors may influence an individual’s decision regarding method in a suicide act, but there is substantial support that easy access influences the choice of method. In many countries, restrictions of access to common means of suicide has lead to lower overall suicide rates, particularly regarding suicide by firearms in USA, detoxification of domestic and motor vehicle gas in England and other countries, toxic pesticides in rural areas, barriers at jumping sites and hanging, by introducing “safe rooms” in prisons and hospitals. Moreover, decline in prescription of barbiturates and tricyclic antidepressants (TCAs), as well as limitation of drugs pack size for paracetamol and salicylate has reduced suicides by overdose, while increased prescription of SSRIs seems to have lowered suicidal rates. *Conclusions*: Restriction to means of suicide may be particularly effective in contexts where the method is popular, highly lethal, widely available, and/or not easily substituted by other similar methods. However, since there is some risk of means substitution, restriction of access should be implemented in conjunction with other suicide prevention strategies.

## 1. Introduction

Suicide is a major public health problem, representing the 10th leading cause of death worldwide [1]. The incidence rate for completed suicide varies considerably between different countries, from 1.1 per 100,000 inhabitants in Azerbaijan to 51.6 per 100,000 inhabitants in Lithuania [2]. The highest suicide rates are found in Eastern European countries (Belarus, Estonia, Lithuania and Russia); low rates are found mainly in Latin America (Colombia, Paraguay) and in some countries in Asia (Philippines and Thailand), while countries in other parts of Europe, in North America, and other parts of Asia and the Pacific tend to fall somewhere in between these extremes. 

Seeking effective intervention strategies for suicide prevention represents an important public health task. Restricting access to means of suicide has been shown to be effective in reducing completion [3], together with strategies aimed to identify and prevent suicidal acts in individuals at risk. In the present paper we examined the empirical and clinical literature on controlling the access to means of suicide. 

A large body of literature sustains that the majority of attempters does not die by suicide. On the other hand, rates of death for suicide increase in subjects with repeated and life-threatening attempts [4]. Intensity of intent may be an important indicator. Studies employing scales to measure suicidal intent, reported intensity of intent to die as a major predictor of suicide completion in suicide attempters [5]. The problem in predicting suicide from ideation, attempts, or other risk factors is the predominance of false positives and some have concluded that, despite much research, there is no possibility to reliably predict and prevent suicide in any individual [6]. Many studies have identified risk factors associated to suicide completion, such as gender, previous attempts, suicidal ideation, a diagnosis of a depressive disorder or schizophrenia, but such predictors failed to identify the patients committing suicide [7]. On the other hand, Mann *et al*. [8] stated that “Suicide prevention is possible because up to 83% of suicides have had contact with a primary care physician within a year before their death and up to 66% within a month. Thus, a key prevention strategy is improved screening of depressed patients by primary care physicians and better treatment of major depression”. Many interventions such as pharmacotherapy and psychotherapy, education of professionals and gate keepers, restricting media coverage and reducing access to means, have been indicated by Mann *et al*. [8] as strategies to reduce the frequency of suicide attempts. 

There is evidence in literature supporting suicide means reduction as an effective preventive strategy [9,10]. In the present review we aimed to summarize methods of suicide attempts and studies addressing and evaluating the possibility to restrict access to such methods as preventive strategies for suicide attempt.

## 2. Methods

Studies included were selected if they addressed (1) suicide means and/or (2) restriction of access to such means. Studies focused on other preventive strategies for suicidal behavior, such as educational programs and treatment intervention were not considered for the present review. A literature search by common databases (PubMed, ISI Web of Science and the Cochrane library) was performed. The following keywords were entered alone or combined with each other in order to detect relevant studies: suicide, suicide attempt, suicidal behavior, suicide method, suicide means, suicide prediction, prevention, hanging, poisoning, toxic gas, pesticides, drugs, overdose, jumping, lying, firearms, drowning, restricting access to means of suicide, suicide means reduction. Studies were also selected from reference lists of previous reviews or other relevant studies. 

## 3. Results

### 3.1. Means of Suicide

Within the context of the European Alliance Against Depression (EAAD) project, an international partnership of 16 European countries, hanging was found to be the most frequent means of suicide (49.5%), followed by poisoning by drugs (12.7%), jumping (9.5%), firearms (7.6%), poisoning by other means (5.1%), jumping or lying before moving object (5.0%), drowning (4.2%). Other methods accounted for 6.3% of remaining suicides [11]. 

A number of factors may influence an individual’s decision regarding choice of the method in a suicide act. Gender differences may play an important role. Indeed, men more often recur to violent and highly lethal methods, and this fact has been hypothesized as the cause of higher rates of completed suicide in males than in females in all European countries [2]. According to EAAD data, men had a higher risk of using firearms and hanging, and a lower risk of poisoning by drugs, drowning and jumping, as compared to women [11]. Notwithstanding gender differences, it has been shown that individuals have a preference for a specific means. Depending on the individual, certain forms of death seem more inculpating or exculpating, or more terrifying, painful or shameful. Methods of suicide may also vary across world regions and cultures. According to the data derived from World Health Organization (WHO) mortality database, poisoning by pesticide is common in many Asian countries and in Latin America; poisoning by drugs is common in both Nordic countries and the United Kingdom. Hanging is the preferred method of suicide in Eastern Europe, as is firearm suicide in the US and jumping from a high place in cities and urban societies such as Hong Kong, China [12]. Media can also increase “cognitive” availability of a particular suicide method by distributing technical information about how to enact the method, sensationalizing it and by giving inaccurate portrayals that may encourage use of a specific method [13]. 

There is substantial support for the suggestion that ease of access influences method choice. For example, overdose survivors indicate that they chose overdose because drugs were readily available in the household; over half of suicides in rural parts of China are by pesticides or rat poison; in the USA, suicides with firearms are committed by people with access to guns. Hawton [14] has argued that means restriction may be effective for suicide methods quickly accessible, particularly in the prevention of high-lethal impulsive suicidal behaviors. An early study by Marzuk *et al*. [15] investigated methods of suicide according to their accessibility in the five counties of New York City. The counties had similar suicide rates involving methods that were equally accessible in each county (e.g., hanging, laceration, suffocation, firearms); while all of the differences among counties were explained by differentially available methods in the counties, principally fall from height, overdose of prescription medications, and carbon monoxide poisoning. Thomas and Gunnell [16] also underlined that the rapid rise in gas suicide deaths in the 1920s demonstrate how quickly a new method of suicide can be established in a population when it is easily available. According to a recent review of the literature on inpatient suicides, the methods used for suicide were linked to availability of means [17]. Stark *et al*. 2011 [18] also emphasized the importance of availability of methods particularly in rural areas, where firearm ownership is common and a large number of country dwellers are familiar with their use, as well as pesticides are often widely available and poorly controlled, particularly in low- and middle-income countries. In the US, household firearm ownership levels are strongly associated with higher rates of suicide, consistent with the hypothesis that the availability of lethal means increases the rate of completed suicide [19]. In an Indian population, not only easy availability of pesticides was a risk factor, but it was also reported that the majority of acts of deliberate suicidal behaviors (>90%) were committed inside the home, where suicidal means may be quickly available [20]. A further study in a west coastal region of India, confirmed that availability, accessibility, popularity, and socio-acceptability seem to be the major determinants in the choice of methods [21].

### 3.2. Restricting Access to Means of Suicide

A potential problem for suicide prevention by limiting access to methods is the substitution hypothesis: if one suicide method is unavailable, it will be replaced with another. It is recognized that suicidal crisis are usually of short duration and that, if their fatal outcome is prevented by help available in the meanwhile, they will not be repeated. If individuals seem to have a preference for a specific means of suicide and if they essentially experience short-lived crises, restricting access to a specific method should not bring about an increase in the substitution by other means [13]. Therefore, the fundamental assumption underlying restricting access to means of suicide is that, in many cases, it may delay an attempt until the period of high-risk passes. Moreover, if access to highly lethal methods of suicide is reduced, even where substitution occurs, the proportion of people who survive suicide attempts will be increased [22]. 

Means reduction can occur on a population or on an individual level. Individual level approach involves limiting access to a particular means for individuals at risk of suicide. Population-level means reduction consists in restriction of means availability by trends or policy changes [10]. In some Countries, restrictions of access to common means of suicide, has lead to lower overall suicide rates. Most of the evidence comes from studies examining the association between a population-level decrease in the availability of a given lethal means of suicide and method-specific suicide rates. 

TOXIC DOMESTIC GAS. One of the early demonstration of the effectiveness of limiting access to means was detoxification of gas in homes, as observed by Kreitman [23] between 1955 and 1975, when the gas in English homes was gradually changed from toxic charcoal gas to non-toxic natural gas. Detoxification of domestic gas has lead to a reduction of annual suicide rate by 19–33% [23]. An examination of suicide rates in Switzerland after domestic gas detoxification indicated a decline not only gas suicide rates, but also overall suicide rates. The same effect was observed after the detoxification of domestic gas in Australia, Japan and the USA. However, in England and Scotland, Netherland and Germany, though a marked decline in suicide due to domestic gas, a slight increase of deaths by other means was observed (see [8]). 

CATALYTIC CONVERTERS IN MOTOR VEHICLES. Sometime after the reduction of suicides in England by detoxification of domestic gas, there was a gradual increase in deaths from car exhaust, suggesting some substitution. On the other hand, the number of cars *per capita* was concurrently increasing in North European countries [24]. In USA, after the implementation of strict controls on emissions in motor vehicle exhaust gas, rates of accidents and suicide by CO_2_ decreased drastically [25]. Similarly, in Britain, between 1990 and 1997, the increasing use of catalytic converters led to a decreased rate of suicide by motor vehicle exhaust gas [26].

The methods of suicide by domestic gas and motor gas may have been relatively invulnerable to substitution because the characteristics of gas poisoning are quite unique and not easily replaced. Gas poisoning was indeed considered to be highly lethal, though painless, non-disfiguring and requiring little planning [3]. On the other hand a striking increase of suicide cases involving helium inhalation between 2005 and 2009 has been recently reported. Given the availability of helium and the recent promotion of this method of suicide, it is quite possible that this may represent a newly emerging trend in suicide deaths [27].

FIREARMS. Suicide rates are distinctly higher in countries with lax gun control [3] and the proportion of households owning firearms is highly correlated with the proportion of firearm suicides [28]. In 1976 the District of Columbia (Washington, DC, USA) adopted a law that banned the purchase, sale, transfer, or possession of handguns by civilians. The adoption of such law coincided with an abrupt decline in suicides by firearms (23%). Moreover, there were no increases in suicides by other methods. In Canada, restrictions to the use of firearms were implemented in 1977, with a decrease over time of total suicide rates and firearm suicide; though a slight indication of substitution of other methods for suicide was observed. Legal restrictions to firearms produced the same reduction in firearm suicide in Australia after 1980, in Queensland after 1992 and 32 states in the USA after 1994 [8]. A re-analysis of suicide rates in USA during the period 1981–2002, showed that the decline in household firearm ownership was associated with significant declines in rates of firearm suicide [29]. Moreover, a significant decline in suicides was seen after the introduction of the Firearms Amendment Act in England (1989) [30] and the enactment of stringent firearm laws in Finland (1997) [31]. A recent study on college and university students in US covering the years 2004–2009 reported lower suicide rates attributable to a nine-fold decrease in the availability of firearms on campuses *vs*. homes [32]. These data suggests that even where the use of firearms is relatively rare, restricting access can have a beneficial effect for particular groups at high risk for suicide. Also, delaying access to firearms may be a helpful, since the risk for suicide by firearms decreases exponentially after more than one week after purchase [33]. All together, these data overall suggest that a decline in firearm ownership is associated with reduced rates of firearm suicides. However, it has been recently reported that regulations that seek to prohibit high risk individuals from owning firearms were proven to have poor effects in the USA, though they have a significant deterrent effect on overall male suicide [34]. A further recent study reported that, though the rate of suicide by firearms, as well as other methods declined markedly, the hanging/suffocation rate increased significantly from 1992 to 2006 among young in the US [35], suggesting that restriction of some means may increase the rate of means that cannot be easily restricted. Nevertheless, it has to be taken into account that there are many different types of state firearm regulations. Some seek to establish general oversight over individuals owning firearms, other laws seek to prevent gun trafficking and the use of firearms in crimes. A number of laws are designed to prevent firearm ownership by individuals considered disproportionately likely to commit gun crimes. In some states, the requirement a “cooling off” period of some specified period before the purchase is aimed to reduce the consequences of impulsive firearm purchases [34]. Therefore, their impact on suicide prevention may differ depending on the regulation adopted in a specific state.

PESTICIDES. Self-poisoning with certain commonly-used pesticides is highly lethal and is the most common means of suicide in many countries [36]. Three quarters of people who ingested pesticides for suicidal purposes used pesticides that were available within the home or nearby [37]. Limitation of access to pesticides has been implemented successfully in Sri Lanka, where the banning of several highly lethal pesticides led to a 50% reduction in suicides. Similarly, in Western Samoa, restriction laws for toxic pesticides were successfully followed by reduced rates of suicides. In Finland, suicide rate by parathion, a highly lethal pesticide and commonly used for suicide in the 1950s, decreased after its availability was restricted, though an increased suicide rate by other methods was observed (see [8]). A recent study by Vijayakumar and Satheesh-Babu [38] confirmed a significant reduction of suicides by pesticides in four villages in the state of Andhra Pradesh in India that had stopped using chemical pesticides in favor of non-pesticide management, as compared to four villages in the same region that continued to use chemical pesticides. Similarly to fire arms, pesticides are regulated by various states, federal and international agencies. Most countries, including the US, regulate the amount of pesticide residue allowed on a given crop, but the amount may differ across countries. Therefore, regulations on pesticides, when adopted, may different across countries (http://npic.orst.edu/reg/index.html).

BARBITURATES. Death from suicidal drug overdose differs from other forms of suicide in that the drugs are often prescribed by the patient’s physician. A study of suicide in Brisbane (Australia) [39] between the years 1956–1973 revealed that there was a sharp rise in the incidence of deaths from barbiturate overdosage, which reached a peak in the mid 1960s. Since then there had been a steady decline in suicide rates from drug overdose and a smaller fall in the rate of other forms of suicide. From the examination of suicide deaths and the prescribing of barbiturates, benzodiazepines and antidepressant drugs between 1962 and 1973, it was hypothesized that fall in suicide rates was due to the better recognition and treatment of depressive illnesses and to the introduction of the safer benzodiazepines in place of barbiturates. In Britain, Australia, Norway and Sweden, the limitation of barbiturate prescribing was followed by a similar fall in deaths from these drugs [8]. 

PARACETAMOL. In the UK in 1973, analgesic preparations containing salicylates and paracetamol caused 17% of hospital admissions for self-poisoning [40]. A study on suicide by poisoning with paracetamol and salicylates in Britain, recorded an important decreased rate of suicide by this means, after legislation limiting the size of packs of medications in 1998 [41]. On the other hand, Morgan *et al*. [42] suggested that decline in paracetamol deaths may part of a wider trend in decreasing drug-poisoning mortality. Indeed, after the legislation in 1998 limiting the pack size of paracetamol sold in shops in UK, other than a decrease in paracetamol-poisoning mortality, fatal poisoning involving aspirin, antidepressants, and to a lesser degree, paracetamol compounds, also showed similar trends. Bateman *et al*. [43] questioned the efficacy of limiting the pack size of paracetamol. By a review of the literature, they indeed concluded that paracetamol pack size limitation, as applied in the United Kingdom, has not actually reduced paracetamol-related death. However, In Ireland, Corcoran *et al*. [44], reported that the withdrawal from the market of prescription-only analgesic compound of paracetamol and dextropropoxyphene (distalgesic), resulted in a significant reduction of intentional overdose (84%). Though a 44% increase in the rate of intentional drug overdoses involving other prescription compound analgesics, the magnitude of this rate increase was smaller than the magnitude of the decrease in distalgesic-related overdoses.

ANTIDEPRESSANTS. In 1987, in Britain, three of the twelve most commonly taken drugs in completed suicide were antidepressants (dothiepin, amitriptyline and imipramine). Antidepressants accounted for approximately 15% of all drug overdoses [45]. The suicide risk for TCAs and neuroleptics increased with their availability, while the risk for antidepressants other than TCAs (Selective Serotonin Reuptake Inhibitors, SSRIs) decreased despite increased availability [24]. More recent studies, demonstrated a marked decrease of suicide rates after the introduction of SSRIs, and significant associations between SSRIs and other new-generation antidepressants with lower suicide rates, while TCAs would be associated with higher suicide rates [46].

BARRIERS AT JUMPING SITES. An effort to reduce suicide rates limiting access to jump sites has also been made. Though there are many potential jump sites, certain locations tend to gain particular notoriety as suicide spots. A study by Beautrais *et al*. [47] investigated the effect of removal of safety barriers from a central city bridge, a known suicide site, in an Australasian metropolitan area in 1996 after having been in place for 60 years. The study clearly showed that removal of safety barriers led to an immediate and substantial increase in both the numbers and rate of suicide at the site. At the opposite, the installation of barriers on the Clifton suspension bridge, Bristol, England, in 1998 halved suicide rates in the area, from 8 to 4 per year. Further, though the number of incidents on the bridge did not decrease, bridge staff reported that the barriers ‘bought time’, making intervention possible [48]. Installation of safety barriers at known suicide sites, such as the Eiffel Tower, Sidney Harbour Bridge, the Empire State Building and the Duke Ellington Bridge in Washington D.C., has been successful in reducing suicides by jumping at these sites [49,50]. Moreover, there may be a low risk of substitution, as suicide attempters who jumped from certain sites often reported choosing this method due to symbolism associated with the specific site [51]. On the other hand, a recent study aimed to determine whether rates of suicide changed in Toronto after a barrier was erected on the Bloor Street Viaduct, reported that although the barrier prevented suicides at the viaduct, the overall rate of suicide by jumping in Toronto remained unchanged [52]. Moreover, Glasgow [53], by an extensive analysis across 3,116 US counties or county equivalents found that while exposure to local landmark bridges was associated with an increased number of suicides by jumping, no positive relationship between these bridges and the overall number of suicides was detected. Therefore, barriers at jumping sites may prevent suicides to some extent, especially when the site is a site frequently used for suicidal purposes and therefore symbolically characterized, or when they make intervention possible [48]. However, overall studies seem to demonstrate that barriers lead to a reduction in the number of suicides by jumping at the site where they are installed, but not to the overall local suicide rate [53].

“SAFE ROOMS” IN INSTITUTIONAL SETTINGS TO PREVENT HANGING. Hanging is the most common means of suicide in both males and females [54]. Restriction to access to this mean of suicide is not possible, as ligature points and ligatures commonly used are universally available. The only exception is represented by suicide in institutional settings (police custody, prisons and hospitals) where only 10% of suicide occur by hanging [55]. In prisons, cell window bars are the suspension point used in nearly half of prison suicides (48%). Other points of suspension are the bed (11%), cell fittings such as lights, pipes, cupboards, sinks or toilets (13%), or the cell door (5%) [56]. In 1989, the Atlas of Health suggested a wide range of changes to cell design and in 2002 the World Health Organization (WHO) has issued guidelines for Prison Officers regarding the prevention of suicide [57]. A description of “safer cellars” in England and Wales can be found in Gunnel *et al*. [55]. Hanging in psychiatric hospital account for around 3% of all deaths by suicide. Nearly one-third of ward hangings are located in the toilet or bathroom. To prevent suicide by hanging in hospital, it has been suggested that high-risk patients should be given or asked to wear clothes that do not need belts and shoes that do not have laces [58].

MEDIA REPORTS OF SUICIDE. In suicide method choice, how accessible something is may play an important role. As stated before, the media can increase cognitive availability of a particular suicide method by distributing technical information and sensationalizing. Studies suggest that reports of suicides are most likely to have an impact when the method of suicide is described in detail, the story is repeated, the suicide features are described dramatically and prominently, and the individual is someone the audience may identifies with. An impressive case is charcoal burning. In the past decades, rates of suicide by this method have increased in some regions. In Taiwan, it has been reported that charcoal burning suicide attempters were more likely to report that their choice of method was influenced by the media, particularly the portrayal of the method as a peaceful way of dying [59]. More common cases are subway suicides. Subway suicides were reported doubled in Toronto, following reports of subway suicides occurring between 1970 and 1971 [60]. However, after a six-month restriction of reporting of subway suicides, the rate of subway suicides returned to baseline levels. Similarly, after an agreement to abstain from reporting on cases of subway suicide in Vienna, a 75% reduction was observed [61]. Overall, media restriction on reporting suicide may lead to reduced suicide rates. Cognitive availability may be also increased by mental images of suicidal behaviour in people who have been suicidal in the past or exposed to suicidal behaviour in family or friends. In these cases, psychological treatment would be helpful in reducing cognitive vulnerability. A cognitive availability can be also increased by technical information on how to carry the suicidal act out through pro-suicide books and sites on the internet. A way to counteract this effect may rely on emphasizing unpleasant details of suicide (particularly pain). Challenging the false beliefs may be helpful in reducing the popularity of some methods [62]. 

## 4. Discussion and Conclusions

Suicide prevention research has several challenges, partly due to the complexity of factors involved in suicidal behaviour. In the last decades, several epidemiological studies reported changes in rates and methods of suicide and the overall observed decline in rates of suicide in most parts of the worlds coincides with a reduction in the availability of lethal methods [34,63,64]. Therefore, strategies aimed to limit the access to means used in suicide is effective and should be an important part of a suicide prevention strategy [10]. Further, it can be implemented quickly and effects measured relatively easily in comparison to other suicide prevention strategies aimed to identify underlying causes of suicidal behaviour and individuals at risk [62]. To date, no consistent evidence has been provided regarding the effect of restrictions in specific subtypes of patients. Violent and high-lethal methods (fire arms and hanging) are more frequent in males while poisoning is more common in females, and quickly accessible means are common in impulsive suicide attempters. However, cultural factors, cognitive availability, symbolic connotation of the mean, its popularity and socio-acceptability, may all have an important role and of the mean. Restriction to means of suicide may be particularly effective in contexts where the method is popular, highly lethal, widely available, and/or not easily substituted by other similar methods [22]. However, restriction of means of suicide does not exclude the possibility of substitution with other more available means in severely distressed patients. Therefore restriction of access to lethal means of suicide should be implemented in conjunction with other suicide prevention strategies. 
